# Integrative Laser Medicine and High-Tech Acupuncture at the Medical University of Graz, Austria, Europe

**DOI:** 10.1155/2012/103109

**Published:** 2012-04-17

**Authors:** Gerhard Litscher

**Affiliations:** Stronach Research Unit for Complementary and Integrative Laser Medicine, Research Unit of Biomedical Engineering in Anesthesia and Intensive Care Medicine, and TCM Research Center Graz, Medical University of Graz, Auenbruggerplatz 29, 8036 Graz, Austria

## Abstract

At the moment, modernization of acupuncture has a high priority. On the traditional side, acupuncture has only recently been awarded the status of Intangible Cultural Heritage by the UNESCO. On the innovative side, high-tech acupuncture is a registered trademark in Austria. Acupuncture has been used for medical treatment for thousands of years. A large number of empirical data are available but the technical quantification of effects was not possible up to now. Using electroacupuncture, needle, or laser stimulation and modern biomedical techniques, it was possible for the first time to quantify changes in biological activities caused by acupuncture. This paper which serves as introduction for the special issue “High-Tech Acupuncture and Integrative Laser Medicine” of the present journal, focuses on the latest innovative aspects that underline the further enhancement and development of acupuncture. Special emphasis is given to new methodological and technical investigations, for example, results obtained from all kinds of acupuncture innovations (e.g., teleacupuncture) and integrative laser medicine.

## 1. Introduction

**Figure 1:**
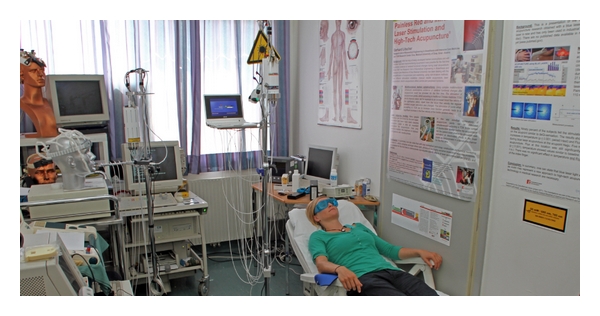
High-tech acupuncture laboratory (2011) at the Medical University of Graz, Austria.

**Figure 2:**
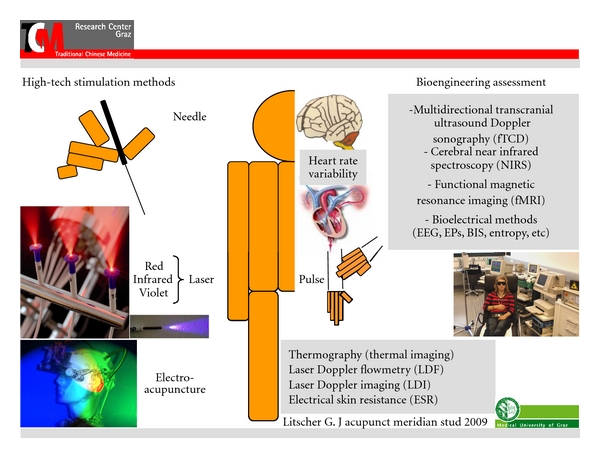
High-tech biomedical methods for manual and computer-based stimulation and quantification of peripheral and cerebral effects of acupuncture. All these procedures are used at the Medical University of Graz, Austria (modified from [12]).

The scientific and technological progress has truly revolutionized eastern and western experimental and clinical medicine recently. The last century was certainly the most innovative phase in medical history. At the Medical University of Graz, our TCM (Traditional Chinese Medicine) Research Center has made various efforts within the last 15 years to modernize acupuncture [1–11], one of the most spectacular Eastern medical procedures. This paper article focuses on the latest aspects that underline the further enhancement and development of modern acupuncture. High-tech acupuncture comprises many different forms of stimulation and recording techniques (G. Litscher, Austrian Patent Office, no. AM 7066/2001, 202 560, valid until 2022) [12]. At the high-tech acupuncture laboratory (see Figure 1) of the Medical University of Graz, a broad spectrum of future-oriented bioengineering methods is used in joint projects with different leading acupuncture institutions in China. One can find an exemplary listing of important noninvasive procedures in Figure 2.

**Figure 3:**
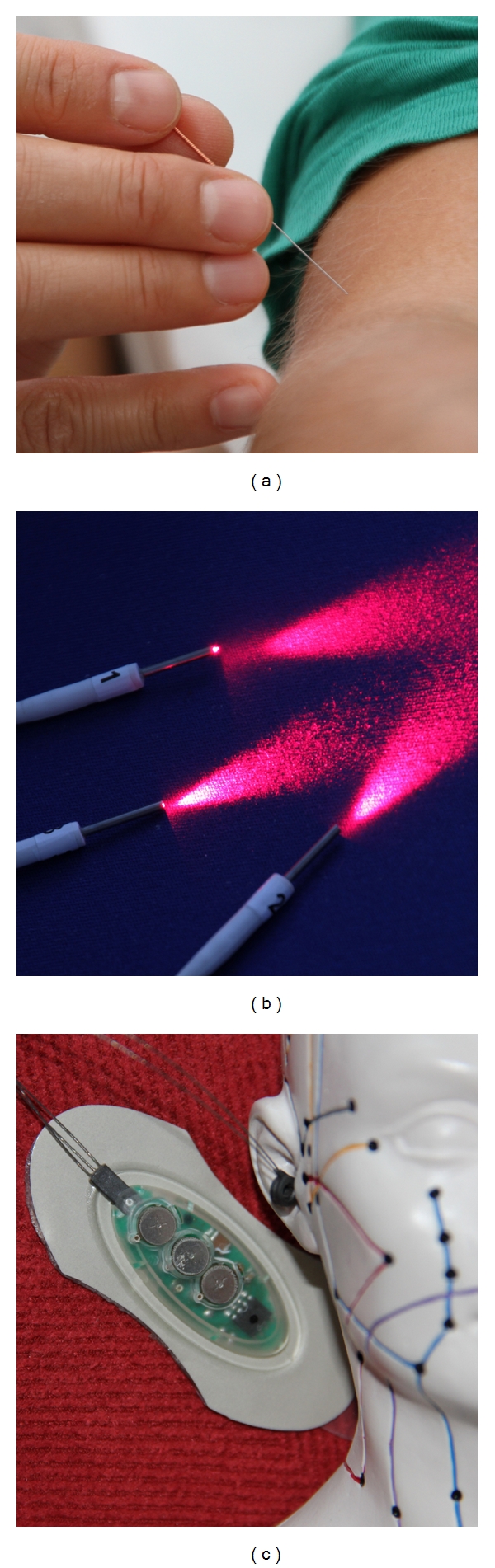
Acupuncture stimulus application: manual needle acupuncture (a), laser needle acupuncture (b), and electrical acupuncture stimulation (c).

For the application of stimuli at the acupuncture points we use manual needle acupuncture (Figure 3(a)), laser needle acupuncture (Figure 3(b)), and electrical methods (Figure 3(c)).

For current acupuncture research, the usage of advanced exploratory tools multidirectional transcranial Doppler ultrasound sonography, cerebral near infrared spectroscopy, functional magnetic resonance imaging, different bioelectrical methods, and other highly sophisticated biomedical equipments, provides revealing insights. The obtained results are absolutely necessary for the acceptance of acupuncture by the western medical community.

## 2. Modern High-Tech Acupuncture Stimulation Methods

Since ancient times, metal needles are the most important tools for stimulating different acupuncture points [13]. New optical and also electrical stimulation methods were scientifically investigated by our research group within the last years. These procedures are described in the following.

### 2.1. Laser Acupuncture (405 nm)

**Figure 4:**
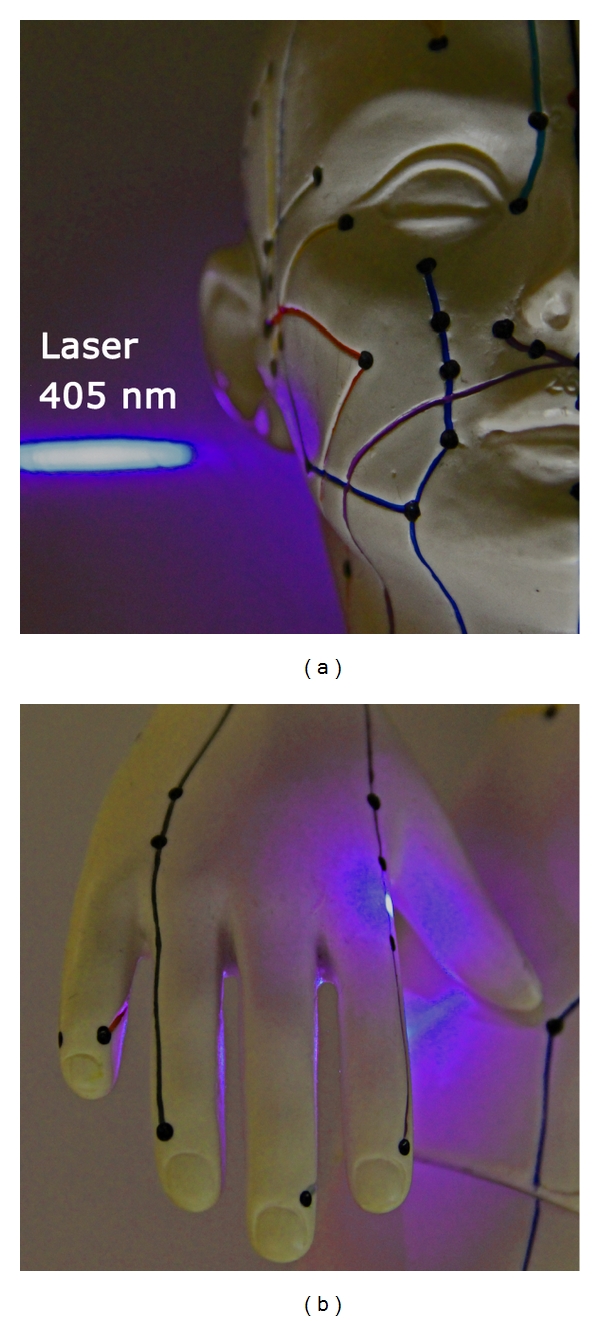
Innovative laser stimulation with a violet laser beam.

Up to now, violet lasers are used only in special areas in medicine [14, 15], because it is a new and still expensive invention. In acupuncture research, violet laser was applied only in a few scientific investigations until now, which were published by our research group [12, 16–20] (Figures 4(a) and 4(b)).

**Figure 5:**
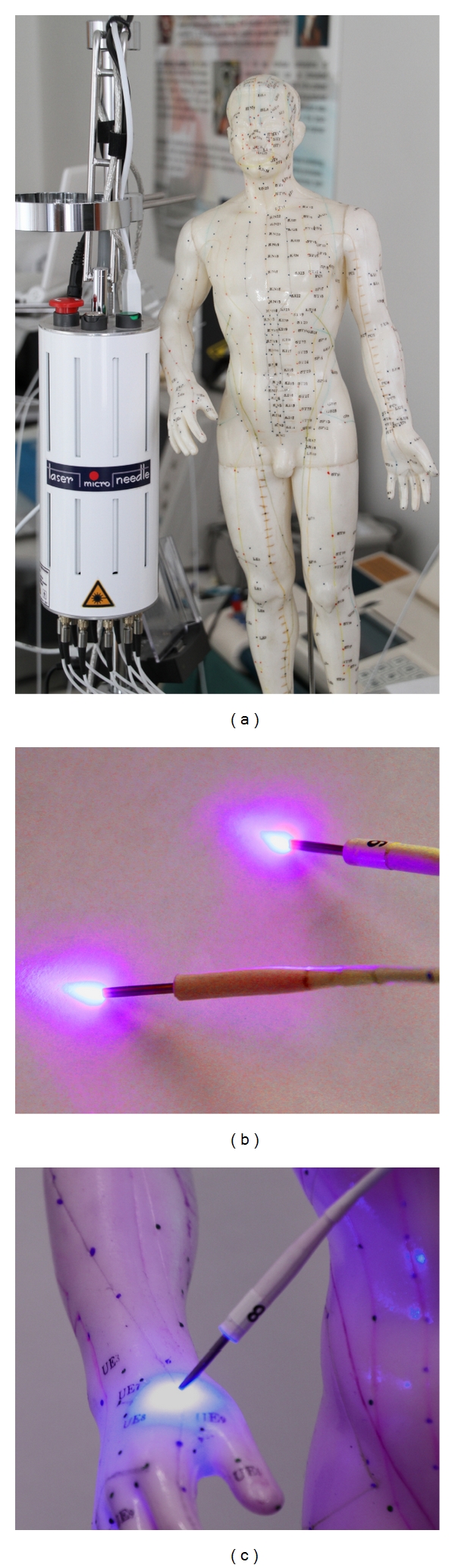
Multichannel system (a) with violet laser needles (b) for acupuncture stimulation (c) used at the Medical University of Graz.

Violet laser needle acupuncture is a new optical method for stimulating different acupuncture points continuously and simultaneously. A wavelength of 405 nm, an output power of 110 mW, and a diameter of 500 *μ*m were used for our experimental investigations. The system consists of 10 semi-conductor injection laser diodes (Figure 5).

Each single needle can emit a different wavelength. We used a continuous wave mode (CW). Due to coupling losses, the output at the tip of the needle is about 100 mW. Irradiation usually lasts 10 min (600 sec), and, therefore, optical power density was very high (range: kJ/cm²) [19]. The violet laser needles are placed vertically at the skin and trigger painless but perceptible stimulation at the acupuncture point.

**Figure 6:**
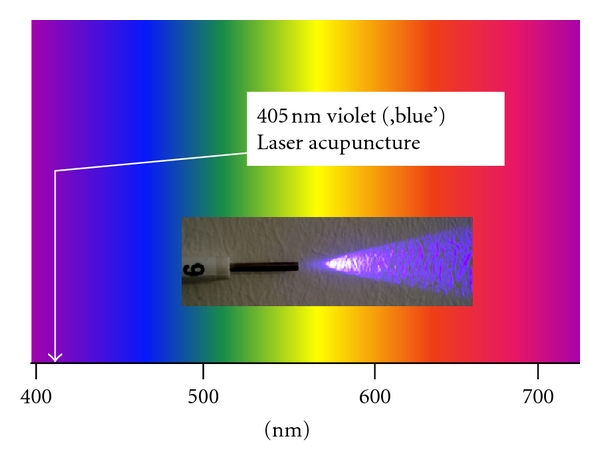
Portion of the electromagnetic spectrum that is visible to (can be detected by) the human eye. Electromagnetic radiation in this range of wavelengths is called visible light. A typical human eye will respond to wavelengths from about 390 to 750 nm. Our violet laser for acupuncture works at a wavelength of 405 nm (modified from [17]).

Violet laser acupuncture was made possible only due to latest inventions. Nakamura et al. [21] developed small, convenient blue and violet lasers which had not been available before. The acupuncture laser equipment used in our studies operates, as already mentioned before, at a wavelength of 405 nanometers. It is worth noticing that this wavelength is not in fact blue but appears to the eye as violet, a colour for which the human eye has a very limited sensitivity (Figure 6).

The violet laser does not have similar penetration depth in human skin as for example the red or infrared laser described in the next subsection (violet: approximately 2 mm versus red/infrared: 2-3 cm [12, 22, 23]); however, there is an evoked deQi-sensation, which is a prerequisite for effective acupuncture stimulation. DeQi is described by patients and volunteers as heaviness or like an electrical current running along the treated meridians. If red (685 nm) or infrared (785 nm) lasers are used, the patients normally do not notice when the laser is started. So in the beginning of the treatment they also do not feel any deQi sensation. Several minutes later (5–10 min) many patients report a pleasant warm and sometimes vibrating feeling in some treated areas [24].

**Figure 7:**
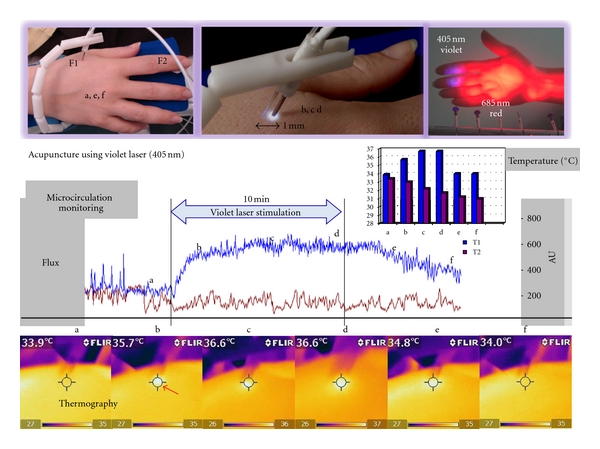
The first violet laser acupuncture using a wavelength of 405 nm. Note the significant increase in microcirculation (Flux1, F1; blue curve) 1 mm beside the violet laser needle at the acupoint Hegu (LI.4). Temperature at this location (T1, same as F1) was measured using thermal infrared imaging (Flir i5). Note also the increase of temperature from 33.9°C to 36.6°C in the region of interest in this volunteer. Flux2 (F2) and temperature2 (T2, location same as F2) were recorded at the index finger (modified from [12]).

In an experimental pilot study we found that violet laser stimulation increases temperature (mean ~1.5°C) and microcirculation (mean ~20%) at the acupoint Hegu (LI.4) significantly and immediately (1 min) after stimulation onset (Figure 7) [12]. The main interesting finding of our second publication concerning violet laser acupuncture was that heart rate decreases significantly within an interval of 5 min after violet laser stimulation onset at the acupoint Neiguan (Pe.6) [16]. Four interesting studies performed recently [17–20] will be presented in the respective subsections in the recording methods section (temperature distribution, microcirculation monitoring, cerebral blood flow velocity, and arterial stiffness and wave reflection).

### 2.2. Laser Acupuncture (685 nm and 785 nm)

**Figure 8:**
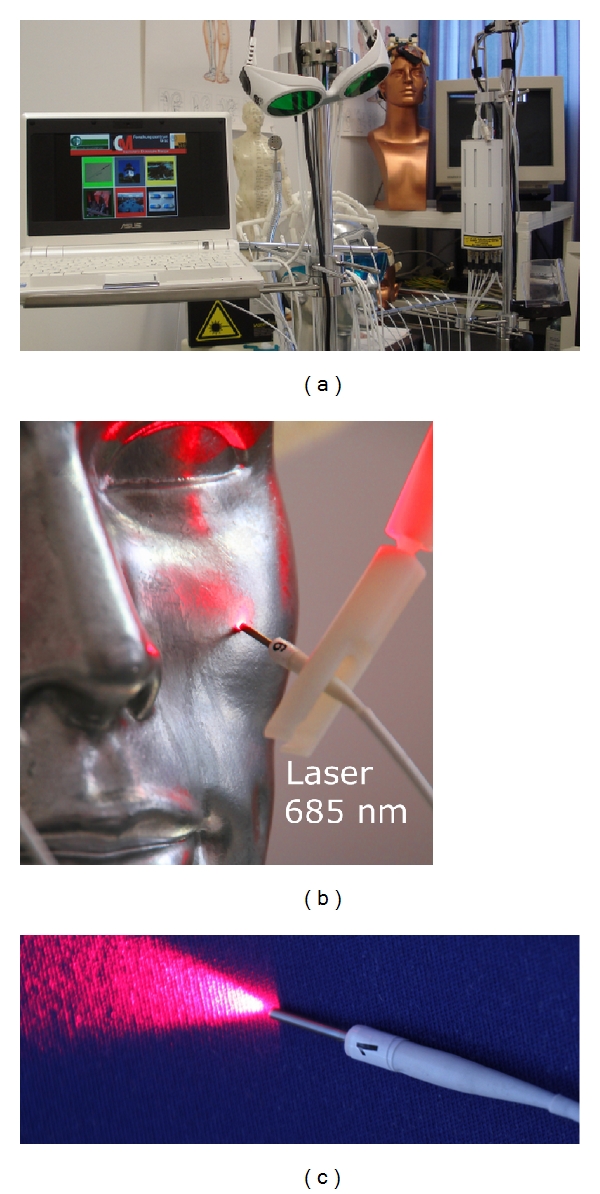
Multichannel laser acupuncture (a) using bichromatic laser needles with red (685 nm) and infrared (785 nm) light (b) and (c).

The first bichromatic laser needles (685 nm and 785 nm) were developed at the University of Paderborn, Germany (Dr. Detlef Schikora), and the first clinical investigations were performed in Lauenförde, Germany (Dr. Michael Weber). The first scientific experiments and publications on this field of research started in 2002 at our Medical University of Graz, Austria [22, 25–29]. A new laser needle acupuncture system based on red and infrared laser light is shown in Figure 8.

Multichannel laser needle acupuncture allows the simultaneous stimulation of individual point combinations [22, 26]. Variations and combinations of acupuncture points according to TCM are possible on the body, or at the ear and hand using Korean or Chinese hand acupuncture. The bichromatic laser needle method is based on systems with 8–12 separate semiconductor laser diodes and emission wavelengths of 685 nm and 785 nm. The system consists of flexible optical light fibers, which conduct the laser light with minimal loss to the laser needle. Thus, a high optical density can be achieved at the distal end of the laser needle. The intensity of the laser needles is optimized in such a way so that the volunteer or patient does not immediately feel the activation of the needle (30–40 mW per needle; diameter 500 *μ*m; duration 10 min; power density ~20 J/cm² per acupuncture point). More details regarding this method are described in previous studies and books [22, 28, 29].

### 2.3. Electroacupuncture

**Figure 9:**
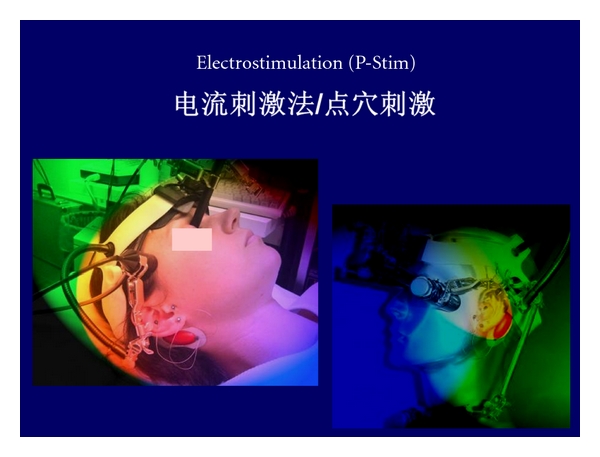
Ear electroacupuncture using a system developed in Austria.

Ear acupuncture can be performed using ultrathin permanent needles (P-Stim, Biegler GmbH, Mauerbach, Austria). A generator located behind the ear produces electrical stimulation impulses that are transferred to the acupuncture points on the ear via the needles (Figure 9).

After selection of the ear acupuncture points, a position tape previously prepared with the P-Stim application pointer is applied. This procedure is repeated until all acupuncture points are marked. Then, the needles can be taken up by the application pointer and applied. The wires are connected to the needles by snapping conductive plastic rings over the needles. Electrical stimulation is performed using a constant AC (alternating) current of 1 mA; impulse duration is 1 ms, stimulus frequency is 1 Hz. This method can be used for pain treatment and was developed by the Medical University of Vienna (P-STIM; Biegler, Austria). This kind of electroacupuncture has also been investigated at our TCM Research Center [30, 31]. In the past, traditional acupuncture and electroacupuncture were characterized by the fact that these methods were confined to the clinic and/or the physicians practice. P-Stim allows continuous, intermitting stimulation up to several days combined with absolute mobility of the patient [32].

Manual needle acupuncture, laser needle stimulation, and electroacupuncture are based on totally different physical principles, and the stimulus intensities are not directly comparable. However, all three methods can induce similar effects in the brain and in the periphery. This has been shown in studies comparing metal needle acupuncture and laser needle stimulation [27] as well as in investigations comparing optical and electrical stimuli [33].

## 3. Modern High-Tech Acupuncture Recording Methods

We have been using many different methods of bioengineering assessment for our investigations over the last years. In this paper, we describe methods for biosignal analysis in the periphery and also for the central nervous system. Heart rate variability measurements and analyses complete the methodological spectrum (cf. Figure 2).

### 3.1. Peripheral Effects

#### 3.1.1. Thermography

**Figure 10:**
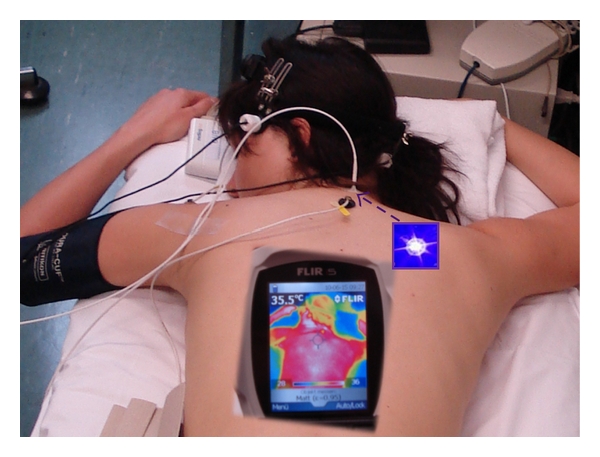
Thermographic monitoring during violet laser acupuncture at the Medical University of Graz. The position of the violet laser needle at the acupoint Dazhui (GV.14) is marked with a violet arrow. Modified from [19].

Thermal imaging involves measurements of the surface temperature using an array of infrared sensors installed in an infrared camera. This imaging allows for the simultaneous measurement of temperatures of multiple points on the skin and is also a reference for the surrounding temperature. For a study performed in our lab recently [19], we used a FLIR i5 infrared camera (Flir Systems Inc., Portland, USA) with a wavelength range of 7.5–13 *μ*m (Figure 10). Temperature distribution measurements were possible in the range between 0°C and +250°C. The data were transferred to a notebook computer using ThermaCAM Researchers Pro 2.8 software (Flir Systems Inc., Portland, USA).

**Figure 11:**
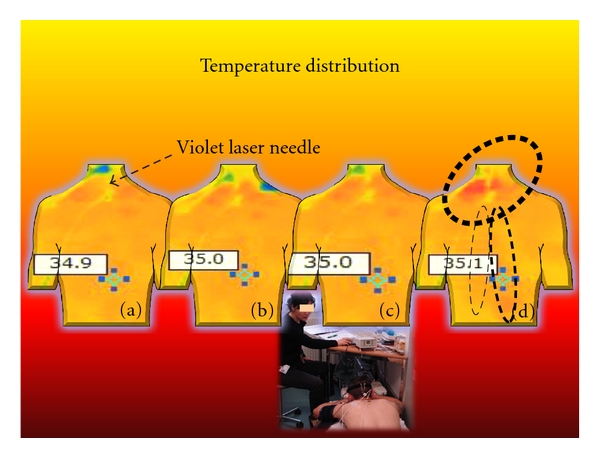
Four thermal images from a 23-year-old healthy male volunteer before (a), during (b) and (c), and after (d) violet laser stimulation at the acupoint Dazhui (GV.14). Note the significant (*P* < 0.05) increase at the shoulder and also the increase in skin temperature at the “far field” location around the Zhiyang (GV.9) area of the same meridian. Modified from [19].

We investigated ten healthy volunteers (mean age ± SD: 24.9 ± 3.3 years; 5 f, 5 m) before, during, and after stimulation using a noninvasive violet (405 nm) laser needle at the Dazhui (GV.14) acupoint. The results showed significant (*P* < 0.001) increases in temperature at the region of interest around the acupuncture point (Figure 11).

In two persons, it was demonstrated that needle acupuncture and placebo (deactivated laser) did not have the same temperature effects [19].

Quantitative thermal imaging is becoming an important method in acupuncture research. Infrared thermography enables the measurement of patients' or healthy volunteers' skin surface temperature profiles without influences caused by direct contact of probes to the skin. Therefore, thermography is also a useful method for evaluating peripheral effects of acupuncture.

There are still methodological limitations of thermal imaging used in acupuncture research. The validity of the method for proving meridian structures according to the view of TCM must be considered critically and analyzed scientifically [34, 35].

#### 3.1.2. Laser Doppler Flowmetry (LDF)

**Figure 12:**
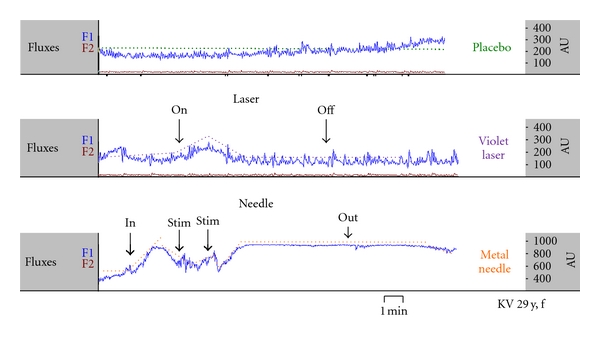
Increase of Flux1 (Dazhui acupoint area) during different kinds of acupuncture stimulation (violet laser and metal needle acupuncture) and during placebo (deactivated laser). Modified from [18].

Using noninvasive laser Doppler flowmetry it is possible to quantify peripheral changes in microcirculation during different methods of acupuncture stimulation [2]. An important parameter is Flux (product of mean blood flow velocity and concentration of red blood cells). The measurements described in Figure 12 were performed with the LDF-monitor DRT4 (Moore Instruments Ltd., Millwey, Axminster, UK). Edge frequencies were 20 Hz and 22.5 kHz. The temperature unit (5−50°C) had a resolution of 0.2°C and an accuracy of 0.5°C. Two standard probes (DPIT, diameter 8 mm, length 7 mm) were used. Flux was recorded at a distance of 3 cm distal from the acupoint Dazhui (Flux 1) and at a control point proximal to the upper arm (Flux 2). The exact position of this control point was 2 cun above LI.14 and 1 cun medially from the line connecting LI.14 and LI.15. The control point was chosen because no application of pressure occurred in this location, and the region of interest for the thermographic measurements is not influenced by the sensor temperature (cf. Figures 10 and 11).

Three measurements of microcirculation parameters in a 29-year-old female volunteer during placebo, violet laser, and metal needle acupuncture are shown in Figure 12. Compared to violet laser acupuncture, stimulation using needle acupuncture enhanced the effect, whereas the placebo procedure (deactivated laser) did not lead to any significant change [18].

LDF is a technique used for investigating changes in microcirculation. It allows objectifying circulation within the microcapillary range without strongly influencing tissue structures. The principle is based on the Doppler shifting of light when light hits moving particles (erythrocytes).

#### 3.1.3. Laser Doppler Imaging (LDI)

Like LDF, laser Doppler imaging (LDI) is also based on the laser Doppler principle whereby data can be obtained directly from the reflected light and is displayed as a color-coded picture showing the distribution of tissue perfusion. LDI is a further advancement of punctual LDF. Here, laser light with low output is directed to the surface of the skin. A servomotor directs the laser light to a maximum of 4096 different positions from one measurement point to the next [36].

The “PIM II” laser Doppler perfusion measurement system from Lisca AB (Linköping, Sweden) includes a scanning head, an optoisolator unit, an AD converter, and a computer. The scanning head comprises a laser unit, an optical system for gradual scanning, and an optode detector unit. The optode isolator unit includes circuits for signal evaluation, stepper motor drive, and circuits to guarantee galvanic isolation between the scanning head and the computer.

A solid laser (670 nm) scans the tissue surface step by step. The reflected light is checked for Doppler effects at 4096 positions of the tissue surface. Monochromatic laser light penetrates each tissue area to a depth of several hundred micrometers. When a moving particle such as a red blood cell is hit, one part of the photons experiences a frequency shift according to the Dopplers principle; the remaining photons in stationary tissues do not underlie these changes. A small part of the reflected light comprises changed and unchanged light and thus influences the photodetector in the scanning head. The combination of light on the photo detector surface results in a change in photo current determined by the Doppler-shifted particles of light. Major Doppler frequency shifts caused by the high velocity of moving blood cells usually increase the frequency of photocurrent, whereas the peak of fluctuation reflects the number of moving blood cells. If this fluctuation element of photo current is guided through a filter and the immediate energy is calculated, a signal for tissue circulation can be derived (i.e., mean velocity multiplied by the concentration of blood cells within tissue distribution).

Any relative movement between laser light and tissue must be avoided during Doppler signal registration in order to prevent artifacts. The system guarantees this with its patented step-by-step scanning method. The laser light remains fixed in relationship to tissue, while the reflected light is analyzed. For registration of light reflection, laser light remains at each single measurement point for about 50 ms.

**Figure 13:**
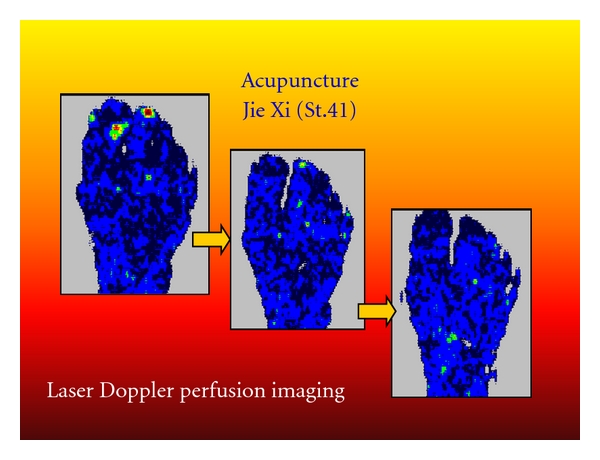
Laser Doppler perfusion imaging of the foot of a 62-year-old patient: red/yellow/green/blue: highest relative perfusion; black: lowest perfusion. Note the increase in mean perfusion (change from black to blue) in the entire region of interest. Modified from [37].

Figure 13 shows a continuous microcirculation monitoring of the right foot in a 62-year-old patient with cryoglobulinaemia before, during, and after manual needle acupuncture at Jie Xi (St.41) acupoint [37]. In addition to visual classification, calculation of alterations of mean perfusion was performed.

These results suggest that the new biomedical method of laser Doppler imaging may be useful to monitor peripheral effects of acupuncture on microcirculation. LDI studies on acupuncture show that needle acupuncture and also laser needle acupuncture can lead to quantifiable changes in microcirculation parameters in addition to the changes in temperature of skin surface.

#### 3.1.4. Electrical Skin Resistance

A scientific literature research (http://www.pubmed.gov/) shows more than 600 articles (January 2012) referring to electrical skin resistance measurements. Most of them are case reports. Only eighteen studies meet the criteria for further evaluation with regard to evidence-based medicine [38, 39]. These studies can be divided into acupuncture studies (*n* = 9) and the so-called “meridian studies” (*n* = 9). Five out of nine acupuncture point studies showed positive association between acupuncture point and lower electrical resistance and impedance. Four studies on this topic showed contrary results. The present limitations of the method are based on the one hand on the small tip of a pen-shaped device, which is pressed manually against the skin by the examiner and on the other hand on the repeated pressing of the electrode tip into the skin or scratching over the skin surface. Therefore an electrical skin resistance mapping consisting of many electrodes is desirable and has already been performed recently by our group and others [39–41].

The newly developed multichannel skin resistance measuring system from our group [40, 41] is used to characterize the variability in electrical resistance measurements in and around an acupoint and a nonacupoint. The system measures the skin resistance at 48 points, both absolutely and continuously. With software developed along with the hardware, both a high-resolution measurement and a graphical presentation of possible changes in electrical resistance in the region of interest are possible [41].

**Figure 14:**
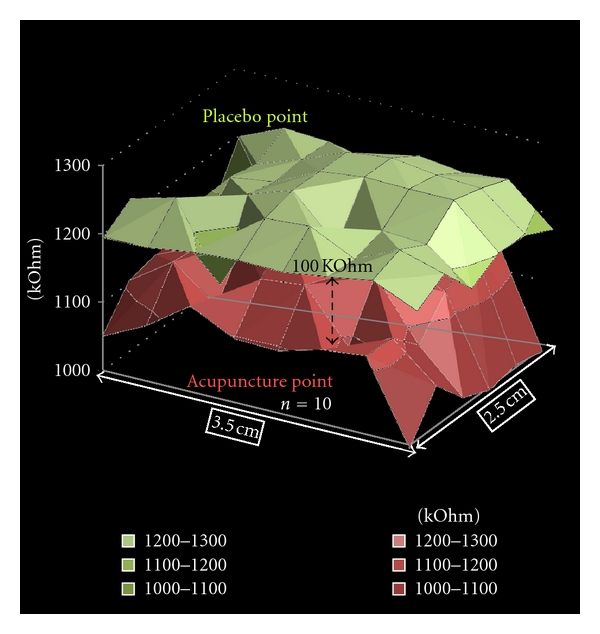
Graphical analysis of 48 channels of electrodermal skin impedance (average values of *n* = 10 persons) at an acupuncture point (below) and a nonacupuncture point (placebo point; above). Note the mean difference between the two surrounding areas is about 100 kOhm. Modified from [42].

Figure 14 shows the results of a study performed in 10 male volunteers, ages 20 to 30 years (mean age ± SD: 24.6 ± 2.5 years) [42]. The aim of this study was to measure the skin resistance of an acupuncture point compared to a placebo point using the new system. The point K$\overset{ˇ}{\text{o}}$ngzuì (Lu.6) and a placebo point on the same level of the acupoint but located on the ulnar side of the heart meridian were used. These points were determined by an experienced acupuncture practitioner. Two measurements were carried out per person. The results of the electrical characterization (skin resistance) of the areas surrounding the acupuncture point and the placebo point were then compared. The measurements of skin resistance at the acupuncture point showed lower impedance values than those taken from the placebo point on the same arm. A significant (*P* < 0.01; ANOVA on ranks) difference of the values was found. Measured values on the acupuncture point were significantly lower (by 106 kOhm; mean values placebo point: 1218 kOhm, mean values acupuncture point: 1112 kOhm) [42].

The electrodermal mapping is an innovative method for highly precise skin resistance measurements [40–42].

### 3.2. Cerebral Effects

#### 3.2.1. Multidirectional Transcranial Ultrasound Doppler Sonography (TCD)

Ultrasound waves are mechanic, matter-bound density waves with frequencies of >20 KHz. These are produced by electric alternating voltage being applied to piezoelectric crystals (“transducer”). The waves propagate in biologic tissue (with the exception of bone) at a nearly constant speed (~1550 m/s). The waves are totally or partially reflected and weakened by scattering and absorption at biologic-acoustic border regions. Ultrasound waves of low intensity (<10 mW/cm²; diagnostics) are considered to be harmless [43, 44].

**Figure 15:**
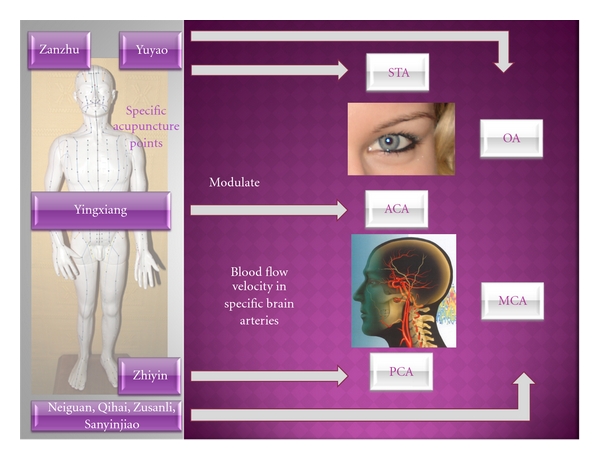
Evidence from multidirectional transcranial ultrasound Doppler sonography: specific acupuncture points (left) modulate blood flow velocity in specific brain arteries (STA supratrochlear artery, OA ophthalmic artery, ACA anterior cerebral artery, MCA middle cerebral artery, and PCA posterior cerebral artery).

Investigations of the neuronal correlates of acupuncture in the human brain were limited by the lack of noninvasive continuous measurement methods such as multidirectional transcranial Doppler sonography in the past. In different studies using these new techniques we have revealed the existence of specific acupoint-brain correlations. Some of these correlations are summarized in Figure 15. For example, acupuncture points traditionally implicated for visual functions were shown to modulate the blood flow velocity of the corresponding cerebral arteries [45–50].

The middle cerebral artery (MCA) with its different branches can usually be investigated at a depth of 3-4 cm, the media main trunk usually lies at a depth of 5.5–6.5 cm. Blood flow velocity in the internal carotid artery (distal part at a depth of 60–65 mm) as well as in the MCA can be determined by transtemporal entry. With increasing depth of measurement volumina a part belonging to the anterior cerebral artery (ACA) can also be determined. If the ultrasound probe is turned slightly in the dorsal and caudal direction, the origin of the posterior cerebral artery (PCA) can be reached at a depth of 65–75 mm.

A restriction of intracranial TCD results could be ultrasound transmission through the skull. According to the literature, particularly older women could only be examined sufficiently in 50 percent of the cases. However, optimal transtemporal registration was not possible in 5–10% of men and younger women either [51].

**Figure 16:**
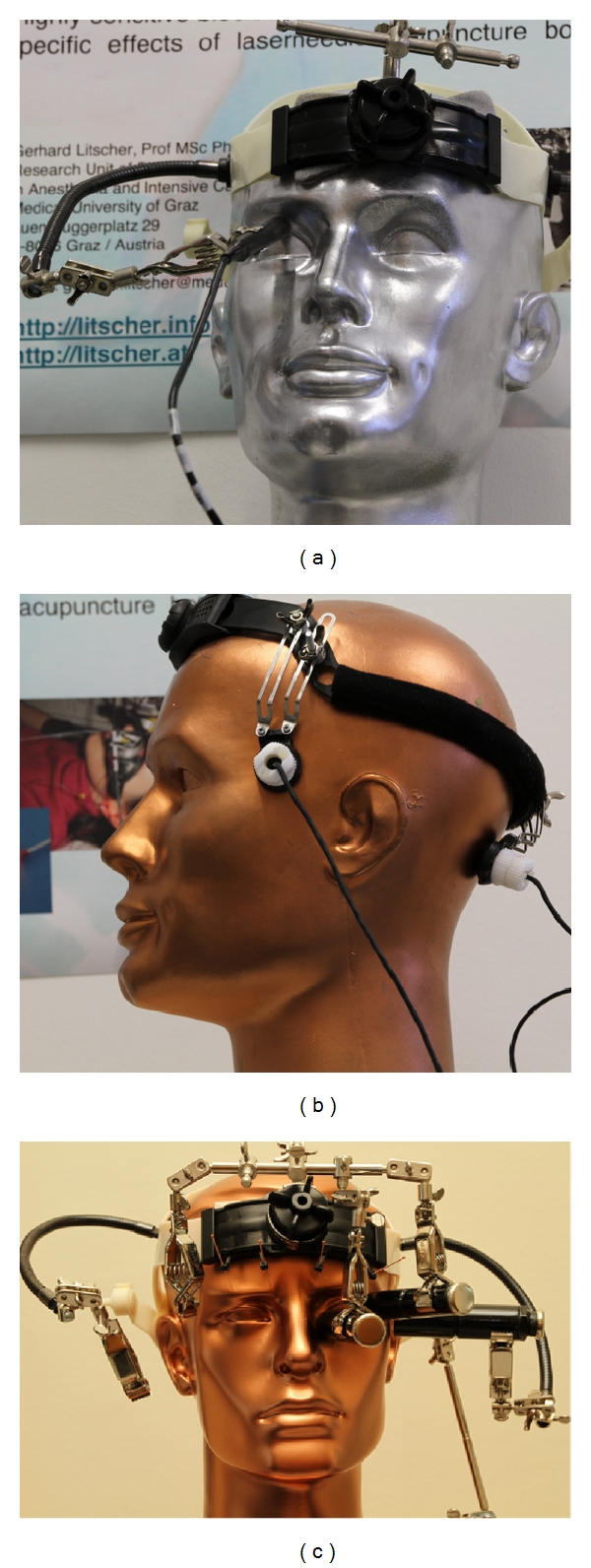
“Windows to the brain” for transcranial ultrasound investigations: transorbital (a) and (c), transtemporal ((b) left, (c) right) and transnuchal ((b) right). The probe holder constructions were developed at the TCM Research Center in Graz, Austria.

In addition to the three transtemporal acoustic windows (front, middle, and rear), transorbital and transnuchal entry for TCD-monitoring is also possible (Figure 16).

Measurement of blood flow profiles in the ophthalmic artery (OA) was performed with a probe, applied lateral to the cornea on the closed bulb of the eye (cf.  Figure 16(c)). Reduction in transmission energy should be achieved as far as possible in order to avoid direct exposure of the lens to sonic waves. A signal from the OA is registered at a depth of 40–50 mm by a probe turned slightly in the central direction [44].

The foramen magnum offers a further path of entry for ultrasound measurements (cf.  Figure 16(b) right). Dependent upon anatomical variations, the basilar artery (BA) lies at a depth between 70 and 110 mm.

**Figure 17:**
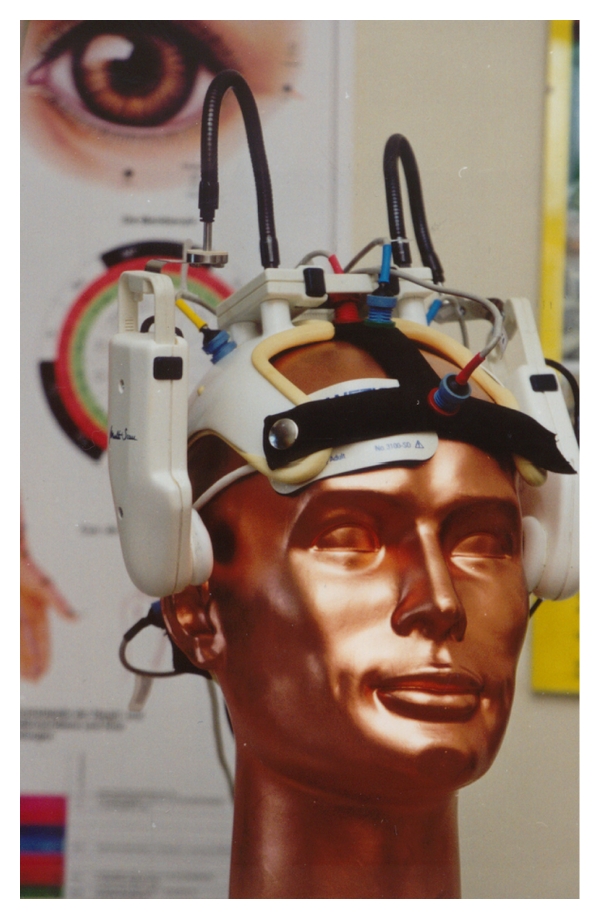
Helmet construction from our research group for the first proof of changes in blood flow velocity in the brain caused by acupuncture. Modified from [45].

For the ultrasound measurements in acupuncture research we have also developed innovative helmet constructions already in the late 1990s (Figure 17) [45–47].

In the year 1997, our interdisciplinary research team was able to scientifically prove that acupuncture needles can increase blood flow velocity in the brain [45]. The computer- and robotic-controlled biosensors and probes integrated in a special measurement helmet, coupled with light, ultrasound and highly sensitive bioelectrical monitoring methods yield reproducible results indicating that the blood flow velocity in the MCA is higher and the oxygen supply in the brain is increased by acupuncture in healthy volunteers [44, 46].

In tests performed with healthy volunteers in 1999, we could prove that acupuncture does not only lead to *general* changes in blood flow velocity in the brain but to *specific* changes (i.e., different regional localization; cf. Figure 15). Thus, stimulation of acupuncture points on the hand or outer side of the foot (e.g., Zhiyin), which according to TCM are often connected to the optical system, leads to an increase in mean blood flow velocity in the PCA, which supplies the occipital center of the brain. At the same time, the blood flow velocity in other cerebral arteries remains nearly unchanged. These effects can only be registered up to now when light stimulation is performed. Comparative investigation of points at the inner edge of the foot did not show changes in blood flow velocity in this particular cerebral artery [48].

Studies with a crossover design using different acupuncture schemes in the same subjects were performed to exclude the placebo effect during acupuncture as much as possible. Each person was treated with an optic acupuncture scheme A, which according to TCM should improve vision, and with another scheme B to improve perfusion in the middle cerebral artery. When using the optic scheme, a significant increase in mean blood flow velocity in the STA and OA occurred, whereas the flow velocity in the MCA remained nearly unchanged. In reverse, scheme B led to a significant increase in blood flow velocity in the MCA with nearly unchanged flow profile patterns in the optic arteries. Several crossover studies were performed and confirm the initial results of selective changes in cerebral perfusion after acupuncture [3, 48–50].

**Figure 18:**
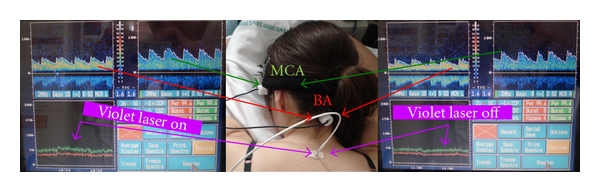
Example of the on/off effect on mean blood flow velocity at the beginning and end of violet laser acupuncture in a healthy female volunteer. Modified from [17].

In a recent study of our TCM Research Center Graz in Austria, which was performed 2010 in [17] in our acupuncture lab, violet laser acupuncture has been investigated in context with blood flow velocity of the BA and MCA (see Figure 18). The aim of that study was to provide selective evidence of specific effects of violet laser acupuncture on mean cerebral blood flow. Again a transcranial Doppler sonography construction was used to monitor blood flow profiles in the BA and MCA simultaneously and continuously. In that controlled study the acupuncture point Dazhui on the upper back was tested with ten healthy volunteers. In addition to an on/off-effect, violet laser stimulation increased the blood flow velocity in the BA significantly compared with the reference interval before laser acupuncture. In the MCA, only minimal, insignificant changes in blood flow velocity were seen. Metal needle acupuncture at the same point intensified the effects; however, blood flow profiles did not change significantly during and after stimulation with deactivated violet laser (placebo) [17].

All our investigations performed with transcranial Doppler sonography demonstrate that acupuncture produces specific and reproducible effects on brain blood flow velocity. However the study designs and also our technology cannot explain the underlying mechanism.

#### 3.2.2. Cerebral Near Infrared Spectroscopy (NIRS)

Near-infrared spectroscopy (NIRS) is a noninvasive optical technique for assessment of functional activity in the human brain [47]. The sophisticated technique uses an optical window in the near infrared (NIR) light spectrum. The first in vivo results in humans were reported by Jöbsis in 1977 [52]. Within a spectral range of approximately 630 to 1300 nm, light can penetrate the cranium and reach sufficient depth to allow investigation of the metabolism in the cerebral cortex. Existing reports on the use of NIRS during acupuncture are mainly focused on one channel recordings. For a review concerning NIRS and acupuncture see Litscher, 2006 [5].

Different instrumental components are used in our NIRS acupuncture research. One system is the NIRO 300 (Hamamatsu Photonics, Hamamatsu, Japan) and another the INVOS 5100 Oximeter (Somanetics Corp., Troy, USA). Tissue oxygenation index (TOI) and regional cerebral oxygen saturation (rSO_2_) were calculated in percent from the proportion of oxygenated and deoxygenated hemoglobin. Normal values lie between 60 and 80 percent [47]. The most important results of our NIRS studies can be summarized as follows [44, 53].

Needling and laser stimulation of a placebo point did not lead to marked changes in cerebral NIRS parameters during or 5 minutes after acupuncture. The combination of Korean hand acupuncture (E2) and Chinese hand acupuncture (Yan Dian) as well as TCM body acupuncture (Zanzhu (UB.2) and Yuyao (Ex.3)) and combined body, ear, and hand acupuncture lead to a marked increase in O_2_Hb and a simultaneous decrease in HHb. This effect was still present 5 minutes after removing the needles or deactivating laser needle stimulation. One case of minimal contrary behavior in O_2_Hb and HHb is present when needling or performing laser stimulation at both ear points (eye and liver). Standard monitoring parameters such as blood pressure did not show any significant changes during all types of acupuncture or combined acupuncture methods.

**Figure 19:**
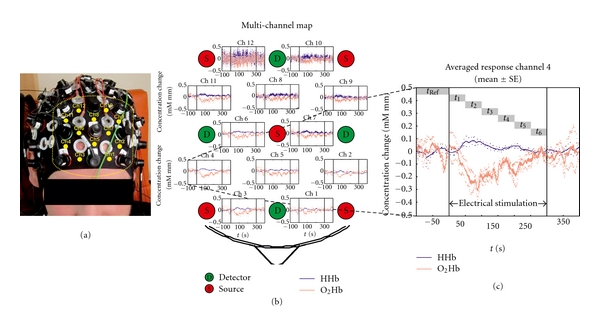
First 50-channel NIRS measurement at the Graz University of Technology (modified from [54]). (a) Electrode cap with detectors and emitters for recording NIRS parameters. The 12 channels presented in the multichannel map in (b) are numbered (Ch1-Ch12). (b) Concentration changes of oxy- (red lines) and deoxyhemoglobin (blue lines) in a map. (c) Averaged responses (mean ± SE (standard error)) of channel 4 before, during, and after electrical stimulation of Battlefield ear acupuncture points.

In a recent study we performed multichannel measurements using an optical neuroimaging system (NIRScout 1624, NIRx Medical Technology, Berlin, Germany) [54]. The system is intuitively operated through a graphical user interface (GUI). The GUI displays measured data for all channels in real time. This system uses wavelengths of 760 and 850 nm and the power is 10 mW per wavelength. The multi-channel system measures the change of O_2_Hb and HHb in the unit of mM mm (m(mol/L)∗mm) and consists of 16 light emitters and 24 photodetectors. The distance between source and detector was 3 cm, and the sampling rate was set to 3 Hz. A special designed electrode cap was used to apply the noninvasive optodes (optical electrodes) above the frontal and motor areas to the intact skull (Figure 19), resulting in a total of 50 measurement channels.

Battlefield acupuncture was developed in the course of researching a more efficient auriculotherapy system for rapid relief of pain [31]. The objective of that study was to investigate possible changes of NIRS parameters in the frontal area of the brain during electrical stimulation of Battlefield acupuncture points. For the first time a 50-channel NIRS recording has been performed to get new insights into the possible cerebral effects of ear acupuncture. Electrical ear stimulation (constant current 1 mA; duration 1 ms, frequency 1 Hz) was used to stimulate the ear points. The main outcome measures were concentration of O_2_Hb and HHb in the brain tissue. Regional decreases of O_2_Hb in the frontal area were found in the 50-channel recordings, reaching their maximum within 100 seconds of stimulation onset [54].

#### 3.2.3. Functional Magnetic Resonance Imaging (fMRI)

**Figure 20:**
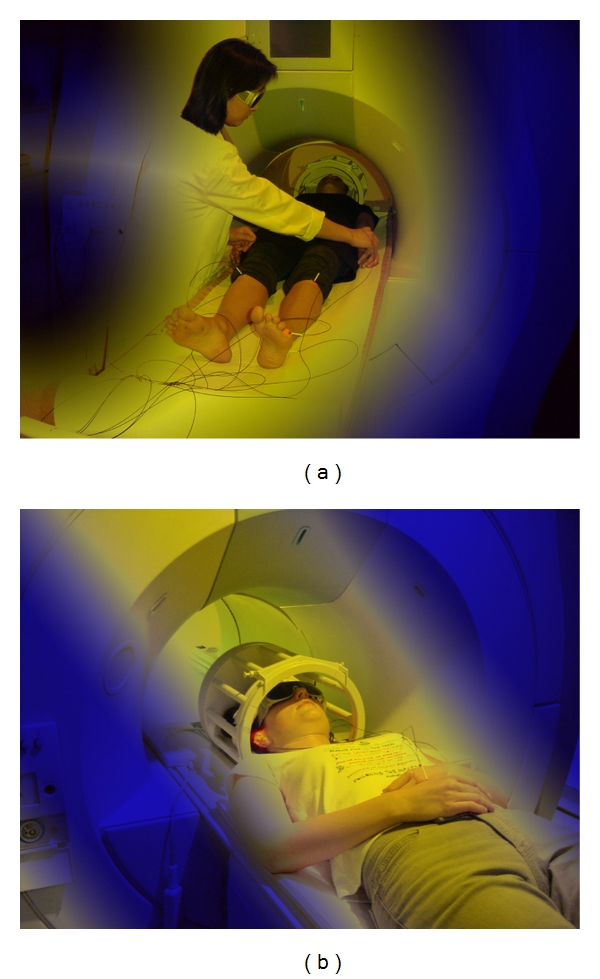
First functional magnetic resonance imaging during laser needle acupuncture at the body (a) and ear (b). These measurements were performed at the Medical University of Graz.

Functional magnetic resonance experiments have also been successfully used to investigate changes in cerebral activity during acupuncture [4, 44, 55] (see Figure 20). The method is based on the indirect representation of neuronal activity and the resulting metabolic and circulatory changes, particularly the relative changes in concentration of oxygenated and deoxygenated haemoglobin [4, 44].

Since the late 1990s, fMRI has been used to investigate the underlying mechanism of the Chinese medical treatment of acupuncture. The research group of Professor Zang Hee Cho from Korea was one of the first to investigate effects of acupuncture using this method [49, 56]. However, some of the authors retracted the paper from Cho et al., 1998, on June 21, 2006. In this retraction they stated that there is no point specificity, at least for pain and analgetic effects.

In the meantime, there are more than 250 publications (http://www.pubmed.gov/; January 2012) on the topic “acupuncture and fMRI.” The point specificity is still discussed controversially. The first review article of fMRI papers was published in Critical Reviews in Biomedical Engineering about five years ago by the author of this contribution [4].

The review by Beissner and Henke published in 2009 [57] should be mentioned especially as it shows clearly the limitations of fMRI investigations in acupuncture research. In a further publication from the same first author [58] the following recommendations are made: standard needles should not be used in MRI; nonferromagnetic metal needles seem to be the best choice for acupuncture points outside of the transmitter coil; only plastic needles are suited for points inside the coil. Laser acupuncture may be a safe alternative, too [4, 22, 58].

Recent studies using fMRI techniques have revealed the existence of specific acupuncture point and brain correlations, for example, acupuncture points traditionally implicated for auditory, visual, sensory, motor, and cognitive functions were shown to modulate the activity of the corresponding cerebral sites [59–63] and also to induce long-lasting effects [64].

#### 3.2.4. Bioelectrical Methods

Neurophysiologic monitoring is gaining more and more attention in acupuncture research [6]. Bioelectrical brain activity can be monitored using bispectral index (BIS) or entropy. Both are numerical descriptors of the electroencephalogram (EEG) and are mainly used for assessing depth of anesthesia.

**Figure 21:**
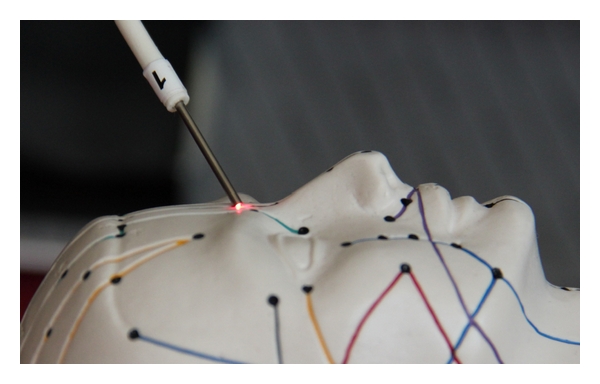
Optical stimulation of the acupuncture point Yintang using a painless laser needle.

In previous studies we have investigated the effects of acupuncture and acupressure at the acupuncture point Yintang in healthy awake volunteers. Acupressure stimulation results in statistically significant and clinically relevant reductions in BIS while needle acupuncture, laser needle acupuncture (Figure 21), and acupressure at a control point resulted in statistically significant but clinically unimportant reductions [65, 66].

**Figure 22:**
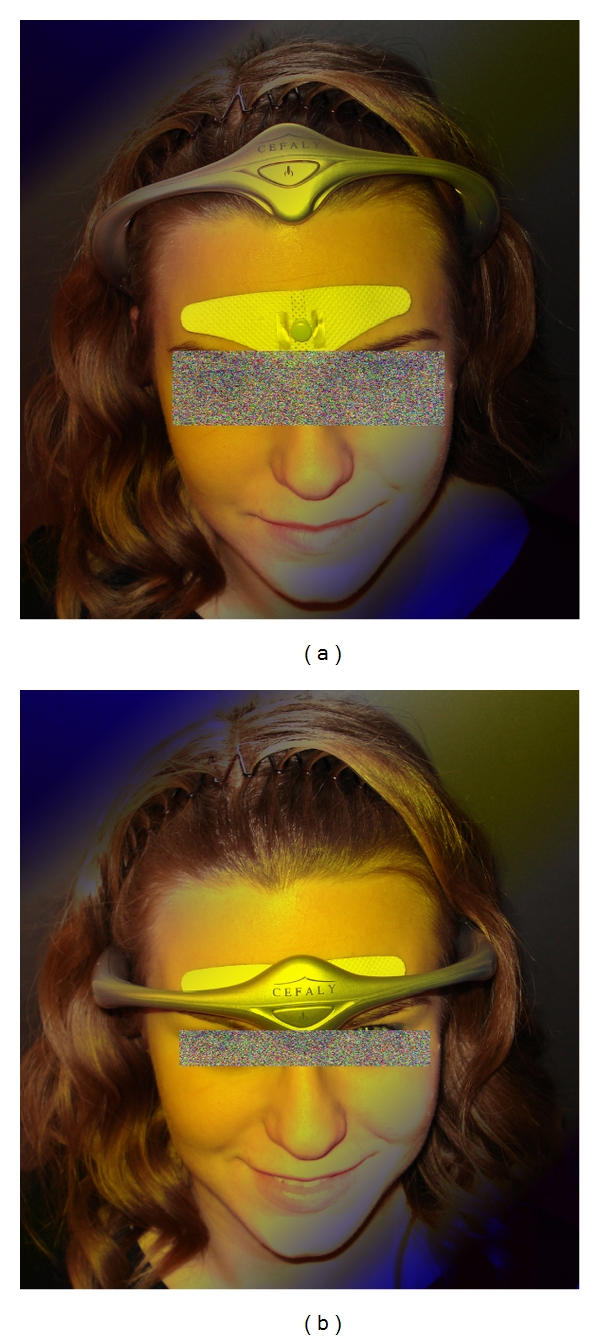
Electrical stimulation at the Yintang acupoint area (light blue point in (a)) at the TCM Research Center Graz. Measurement position (b). With permission of the healthy volunteer and modified from [44, 67].

Another method for stimulating the Yintang area is the application of electrical stimulation (Cefaly, STX-Med, Liege, Belgium) on the forehead (constant current: max. 30 mA; duration of rectangular electrical impulses: 100–500 *μ*s; max. frequency: 150 Hz; stimulus duration: 20 min). This kind of stimulation can induce a state of deep relaxation and sedation as shown in our preliminary studies [67, 68] (Figure 22). The results highlight the electroencephalographic similarities of nonpharmacologically induced sedation and anesthesia.

EEG entropy is a parameter that is mainly used for quantification of depth of anesthesia and sedation. We applied this method of EEG entropy in an acupuncture study. Evidence regarding an acupuncture scheme consisting of “sedative acupuncture points” compared to an acupuncture scheme that is considered to “activate energy” was validated [27]. The investigation was performed in a group of healthy volunteers to determine whether manual needle acupuncture and laser acupuncture stimulations applied to two groups of acupuncture points (sedative versus enhancement of energy) have effects on either state entropy (SE) or response entropy (RE) or both.

A cross-over design was used. This means that two different acupuncture schemes were tested in the same volunteer on different days. The first acupuncture scheme (A) contained the so-called sedative points and included the following acupuncture points: Shenting (Du.24), Yintang (Ex.1), Sedative point 1 (Ex.8, Anmian I), Sedative point 2 (Ex.9, Anmian II), and Shenmen (He.7). Points Shenting and Yintang and their cerebral effects are well documented in the scientific literature [66]. Sedative point 1 is located at the median point of the connecting line between SJ.17 (Yi Feng) and Yi Ming (Ex.7), and sedative point 2 lies at the median point of the connection line between GB.20 (Feng Chi) and Yi Ming (Ex.7). The main indication for the latter two points is sleeplessness. Acupuncture scheme two (B) consists of points that according to TCM should support the “general availability of Qi-energy”: Neiguan (Pe.6), Qihai (Ren 6), Zusanli (St.36) and Sanyinjiao (Sp.6). This scheme has been investigated by our study group and it also leads to an increase in blood flow velocity of the middle cerebral artery [3].

**Figure 23:**
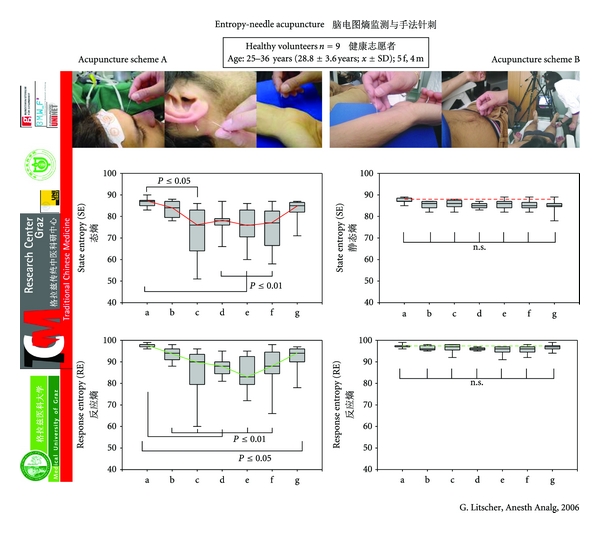
EEG entropy during needle acupuncture. Box-plot illustration of alteration in state (SE) and response (RE) entropy values in nine healthy volunteers 2 min before (a), during ((b) immediately after applying the needles; (c) 2 min after (b); (d) 5 min after (b); (e) 10 min after (b); and (f) 15 min after applying needles), and 2 min after (g) needle acupuncture using two different acupuncture schemes (A and B). Note the significant decrease in entropy when using scheme A (sedation points). The horizontal line in the box indicates the position of the median. The ends of the bars define the 25th and 75th percentile and the error bars mark the 10th and 90th percentile. Modified from [27].

The study shows that two different acupuncture schemes can influence different parameters in the brain. The so-called “sedation point scheme” (A) showed a significant decrease in two entropy parameters (state entropy and response entropy) which can be interpreted as a sedation effect in the EEG (Figure 23).

**Figure 24:**
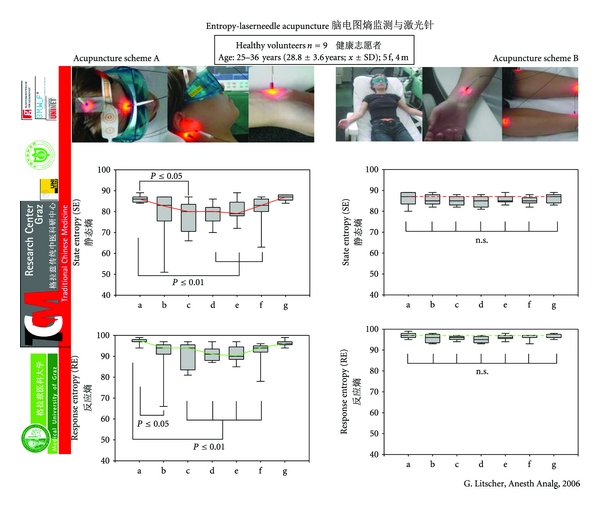
EEG entropy during laser needle acupuncture. Box-plot illustration of changes in state (SE) and response (RE) entropy values in nine healthy volunteers (a) before, (b)–(f) during, and (g) after laser needle acupuncture using two different acupuncture schemes (A and B). For further explanations see Figure 23. Modified from [27].

In the same volunteer, a different acupuncture scheme (B), which according to TCM leads to a general increase in Qi-energy, did not yield this effect. Moreover, several preliminary tests showed that the latter scheme leads to a significant increase in blood flow velocity in the middle cerebral artery [6]. In addition, this study [27] proves that laser needle acupuncture is needle equivalent regarding the values of entropy described (Figure 24).

Electrophysiologic monitoring has been utilized to minimize neurological morbidity from operative manipulations and for monitoring during anesthesia and in the intensive care unit. One goal of such monitoring is to identify changes in brain, spinal cord, and peripheral nerve function prior to irreversible damage. However, neurophysiologic monitoring has also been effective in investigating effects of acupuncture, which may help to discover the complex mechanism.

### 3.3. Autonomic Nervous System Effects

In the last years, several animal [69] and human [7] experimental studies showed that acupuncture can influence the autonomic nervous system (ANS).

#### 3.3.1. Heart Rate Variability (HRV)

Already in the third century the Chinese medical doctor and scientist Wang-Shu Ho realized that variable heart beats are a sign of good health. He stated: “If the pattern of the heart beat becomes regular as the tapping of woodpecker or the dripping of rain from the roof, the patient will be dead in four days.”

Today innovative research including the latest recording technology and also artificial intelligence techniques are used for data acquisition and data analysis of heart rate variability (HRV) in acupuncture research.

HRV is an index value of the neurocontrol of the heart and is measured as the percentage change in sequential chamber complexes, the so-called RR intervals, in the electrocardiogram (ECG). The registration of HRV is performed using three electrodes on the chest. It is important to simultaneously record respiration and if possible continuous blood pressure. The RR intervals in the ECG are controlled by the blood pressure control system, influenced by the hypothalamus and in particular controlled by the vagal cardiovascular center in the lower brainstem. HRV can be quantified over time using registration of percentage changes in RR intervals in the time domain as well as the changes in the frequency range by analysis of electrocardiographic power spectra [7, 44].

HRV parameters are recommended by the task force of the European Society of Cardiology and the North American Society of Pacing and Electrophysiology and are standard since 1996 [70].

**Figure 25:**
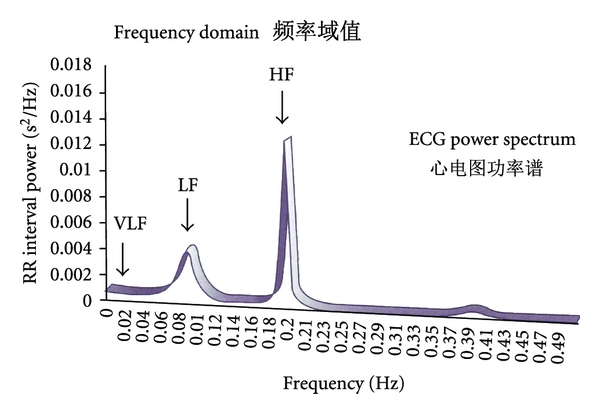
RR interval power spectral density (very low frequency (VLF) band, low frequency (LF) band, and high frequency (HF) band). Modified from [44].

ECG power spectral analysis is thought to provide an understanding of the effects of sympathetic and parasympathetic systems on HRV. Early work pointed out a few bands in the spectrum of HRV that could be interpreted as markers of physiological relevance. Associated mechanisms are thermoregulation which can be found in the very low frequency band (the so-called VLF band), blood pressure (LF band) and respiratory effects (HF-band) (Figure 25). In addition, total power of the heart rate spectrum is calculated for total HRV, and as an important parameter the ratio of the low frequency and high frequency band has become accepted [7, 44, 70].

The so-called “Fire of Life” analysis is a totally new method of visualization of HRV. A low frequency component at around 0.1 Hz is represented by the LF band between 0.05 and 0.15 Hz. The power output in the low frequency band is partly dependent on the sympathetic tone because of baroreceptor activity. Blood pressure waves of third order prove the connection of this so-called 10-second rhythm.

A high frequency component, which is represented by the HF-band, generally between 0.15 and 0.5 Hz, is associated with the breathing frequency and considered an indicator of vagal activity.

**Figure 26:**
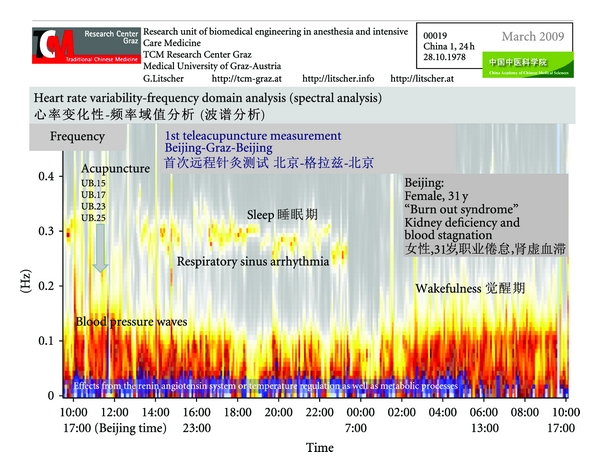
The first teleacupuncture measurement between Europe (Graz) and China (Beijing). The picture shows sympathetic and vagal activity and this can be used to indicate the patient's health and quality of sleep. Acupuncture points: Xinshu (UB.15), Geshu (UB.17), Shenshu (UB.23), and Dachangshu (UB.25). Modified from [71, 72].

In Figure 26 the first results from a teleacupuncture pilot study together with the China Academy of Chinese Medical Sciences in Beijing are presented. The success of the manual needle acupuncture therapy can be quantified using modern technology. Up to now (January 2012), more than 150 teleacupuncture experiments have been successfully performed by our group between China and Austria [71–75].

A 24-hour ECG was recorded using a system partly developed in Austria. The raw data were transferred using internet to the TCM Research Center Graz in Austria from the patients' bedside computer in Beijing to the control computer in Graz over a distance of 7,650 km. Data analysis of different parameters of heart rate and HRV was performed immediately for control of possible therapeutic effects of acupuncture. The acupuncturists in China were informed about the findings immediately and the success of the therapy could be demonstrated objectively. This could probably be useful under special circumstances, for example, for cooperations between experts from different continents as demonstrated in our Sino-Austrian collaboration [44, 76–81].

**Figure 27:**
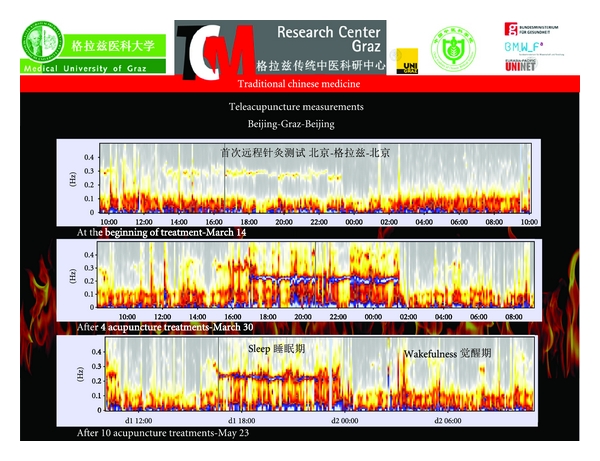
Follow-up measurements during a total of ten acupuncture sessions in China. Note the appearance of an obvious sleep-wake-cycle already after the fourth acupuncture treatment (modified from [72]).

Follow-up measurements demonstrate the success of acupuncture therapy. In this Chinese female patient an obvious sleep-wake-cycle appears during 10 acupuncture treatments within a time period of about nine weeks. The initially reduced “Fire of Life” starts to burn more brightly already after the fourth acupuncture treatment (Figure 27).

**Figure 28:**
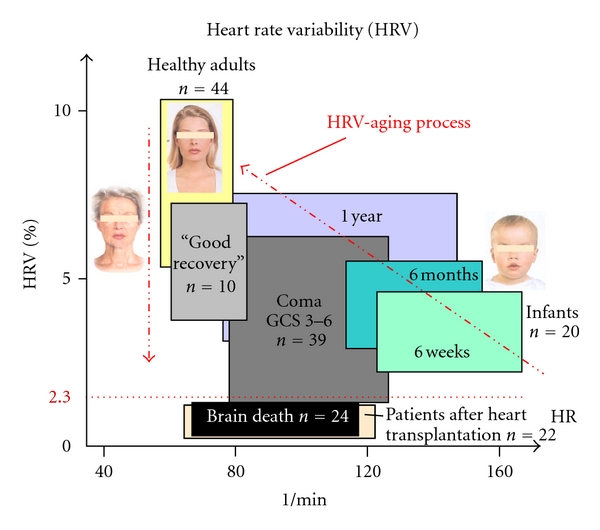
Heart rate (HR) and heart rate variability (HRV) and the aging process.

Heart rate variation depends on age (Figure 28), and there are intraindividual and interindividual variances. Apart from age, circadian variations (sleep-wake-cycle), physical condition and mental and physical exertion are important influencing factors. HRV can also be affected by diverse conditions such as age-related diseases like diabetic neuropathy, renal failure, essential hypertension, cardiac disorders, coronary artery disease, or intracranial lesions [7, 44, 70]. In all cases, different medications have to be taken into account.

HRV can be used as a reliable indicator of the state of health. However, it could be demonstrated that in special syndromes like stress one can counteract this process using different preventive methods like acupuncture. This has been shown in recent investigations concerning patients with burn-out syndrome as performed in common teleacupuncture studies between China and Austria [44, 71–75].

#### 3.3.2. Blood Pressure, Pulse Wave Velocity, and Augmentation Index

**Figure 29:**
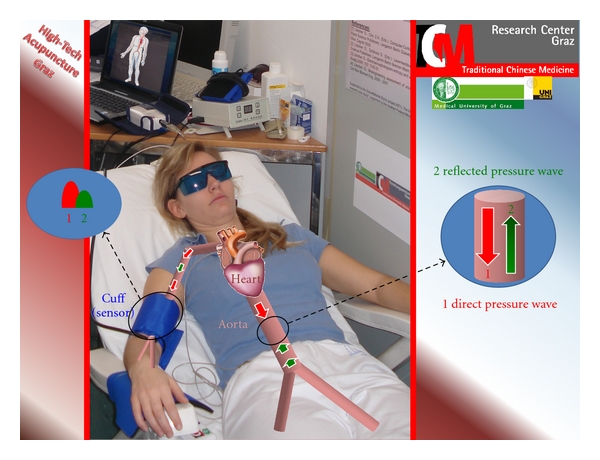
Measurement of the pulse wave velocity and the augmentation index using a cuff at the brachial artery in the lab of the TCM Research Center Graz at the Medical University of Graz. With permission of the volunteer and modified from [20].

In a recent study [20] we investigated the effects of violet laser acupuncture (see stimulation methods) on arterial stiffness and other important parameters of the functional state of heart such as the augmentation index (AIx). Pulse wave velocity (PWV) is a direct marker of arterial stiffness and the AIx one of wave reflection. Both parameters can be registered with a cuff applied to the brachial artery (Figure 29).

The first results from a study in 10 healthy volunteers showed a marked but not statistically significant decrease in PWVaortic and an increase in AIx brachial during and after laser acupuncture at the acupoint Baihui [20]. Further studies including control measurements are necessary.

**Figure 30:**
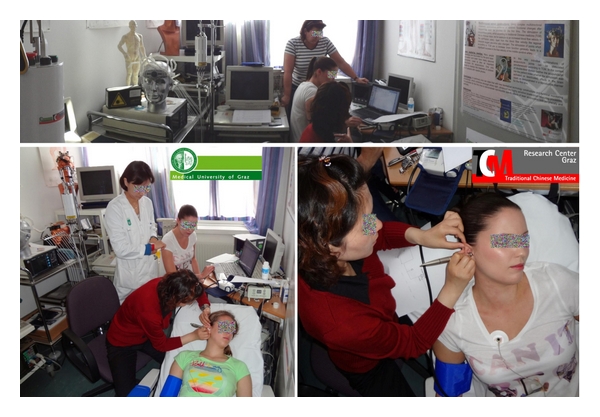
Investigation of ear acupuncture using a new system developed at the TCM Research Center in Graz at the Medical University of Graz (with permission of all medical doctors and volunteers). Modified from [80].

The goal of a further study [80] at our TCM Research Center Graz was to develop a new system for ear acupressure (vibration stimulation) and to perform pilot investigations on the possible acute effects of vibration and manual ear acupressure on HRV, PWVaortic, and the AIx using new noninvasive recording methods (Figure 30).

Investigations were performed in 14 healthy volunteers before, during and after acupressure vibration and manual acupressure stimulation at the “heart” auricular acupuncture point. The results showed a significant decrease in heart rate and a significant increase in total HRV after ear acupressure. The PWV decreased markedly (yet insignificantly), whereas the AIx increased immediately after both methods of stimulation. The increase in the low-frequency band of HRV was mainly based on the intensification of the related mechanism of blood pressure regulation (10-s-rhythm). Further studies in Beijing using animal models and investigations in Graz using human subjects are already in progress.

## 4. Concluding Remarks

Traditional Chinese Medicine (TCM), especially acupuncture, has made many important contributions to the medicine of the world. Using needle, laser needle, electrical stimulation, and modern biomedical recording techniques, changes in the brain and periphery can be quantified.
